# Assessment of Children and “Young” Adult Patients’ Quality of Life after Prosthetic Treatment of Disorders of the Craniofacial Region—A Retrospective Study

**DOI:** 10.3390/jcm13020339

**Published:** 2024-01-07

**Authors:** Elżbieta Wojtyńska, Bohdan Bączkowski, Mariusz Cierech, Elżbieta Mierzwińska-Nastalska

**Affiliations:** Department of Prosthodontics, Medical University of Warsaw, 02-091 Warszawa, Poland; ewojtynska@wum.edu.pl (E.W.); bbaczkowski@wum.edu.pl (B.B.); emierzwinska@wum.edu.pl (E.M.-N.)

**Keywords:** quality of life, dental prosthesis, child preschool, child, adolescent, young adults

## Abstract

Background: Prosthetic rehabilitation for children and juvenile patients with congenital or acquired craniofacial disorders is the area of activity of many clinicians and is a major diagnostic and therapeutic challenge for dentists. Methods: Studies were carried out on a group of 30 patients (10 female and 20 male) aged 2.5 to 30 years who were treated prosthetically due to congenital and acquired craniofacial disorders. The aim of this study was to assess the quality of life using the CPQ 11-14 (Child Perception Questionnaire 11-14), OQLQ (Orthognathic Quality of Life Questionnaire), and PIDAQ (Psychosocial Impact of Dental Aesthetics Questionnaire). Results: Before the prosthetic treatments, the mean values in the individual questionnaires were, respectively, CPQ 11-14—54 points; OQLQ—44.4 points; and PIDAQ—47.6 points. The following values were obtained after the treatments: CPQ 11-14—12.43 points; OQLQ—22.27 points; and PIDAQ—34.03 points. All obtained differences were statistically significant. Conclusions: The total numerical results obtained in all questionnaires decreased, which means that prosthetic rehabilitation had a positive impact on the assessment of the quality of life based on individual categories.

## 1. Introduction

Prosthetic rehabilitation for children and juvenile patients with congenital or acquired craniofacial disorders is the area of activity of many clinicians and is a major diagnostic and therapeutic challenge for dentists [1,2]. Due to the etiopathogenesis, clinical symptoms, the degree of tissue deformation, and the intensive development of the masticatory system, it is a complicated and often multi-stage process [3,4]; considering that the stomatognathic system is a morphological and functional complex of tissues and organs constituting a functional whole, which is involved in food intake, chewing, swallowing, articulating sounds, breathing, expressing emotions, and managing stress, the impact of the diagnosed abnormalities on all structures and functions of this system should be considered [5,6]. Developmental age patients with congenital abnormalities, such as ectodermal dysplasia (DE), hypodontia, oligodontia, cleft lips and/or palates, or with a distorted facial appearance, must not only face functional disorders and altered speech, but also the effect of stigmatization due to the disease. It often changes their appearance, causes spurn by their peers, and affects and lowers their self-esteem [7,8]. In a holistic approach to the treatment of patients with various diseases, in addition to the analysis of objective clinical parameters, more and more attention is being paid to the subjective feelings of patients regarding the impact of the disease on their well-being, their ability to function in a social group or the feeling of exclusion, and the impact of therapy on the quality of life [7,9,10]. The links between the masticatory system and other systems and the impact of its dysfunction on the development and health of an individual are also much more often emphasized. An example is the cybernetic concept of Slavicek, which emphasizes the influence of environmental factors on the functions and disorders of the stomatognathic system and the connection of this system with the brain functions and the patient’s psyche, or the theory of allostasis and the protective role of the masticatory organ according to Sato [11,12].

For years, there have been discussions about the influences of beauty and physical appearance on interpersonal contacts and the evaluation and perception of a person by others. Many researchers have stated that pretty children with nice faces are perceived by the environment as smarter and more socially attractive [13]. According to both sociologists and psychologists, an attractive external appearance greatly influences the development of interpersonal contacts, a positive self-esteem, a strong position in a social group, and a good perceived social well-being [14,15]. According to the definition by World Health Organization (WHO), quality of life (QoL) is a subjective assessment of an individual’s life situation in relation to the culture in which they live and their system of values, goals, expectations, and interests [16]. In the case of patients with chronic diseases or congenital defects, the definition of health-related quality of life (HRQoL) is more often used, where the impact of the disease and its effect on the quality of life and functioning of an individual is assessed. In patients with craniofacial development disorders, the quality of life is assessed in relation to oral health-related quality of life (OHQoL), in which the degrees of influence of the condition of the oral cavity and the appearance of the surrounding tissues on the life of the individual are determined [17,18]. It is assumed that the proper condition of the oral cavity and related tissues is determined by the clinical situation, which is when the development of the stomatognathic system and surrounding tissues is normal and corresponds to the norm for a given stage of developmental age. At the same time, this state enables a person to articulate speech correctly and chew, swallow, and ingest food without restrictions on consistency [19,20,21,22].

The aim of this study was to assess the quality of life of patients of developmental age and “young” adults treated prosthetically due to congenital and acquired craniofacial disorders using three different questionnaires.

## 2. Materials and Methods

### 2.1. Patients

Questionnaire studies on the assessment of the quality of life were carried out in a group of 73 patients, of which 30 (10 female and 20 male) aged 2.5 to 30 years had complete answers included in the questionnaires both before and after prosthetic treatment due to congenital and acquired craniofacial disorders. Children and young adults with incompletely filled questionnaires or taking any kind of medications that may affect their quality of life were excluded. All of the patients signed the contest statement and were informed that the results without personal data may be used in further publications. All patients were informed that filling questionnaires was voluntary and lack of consent to complete the survey would in no way affect further therapeutic procedures and the patients’ treatment plans.

**Figure 1:**
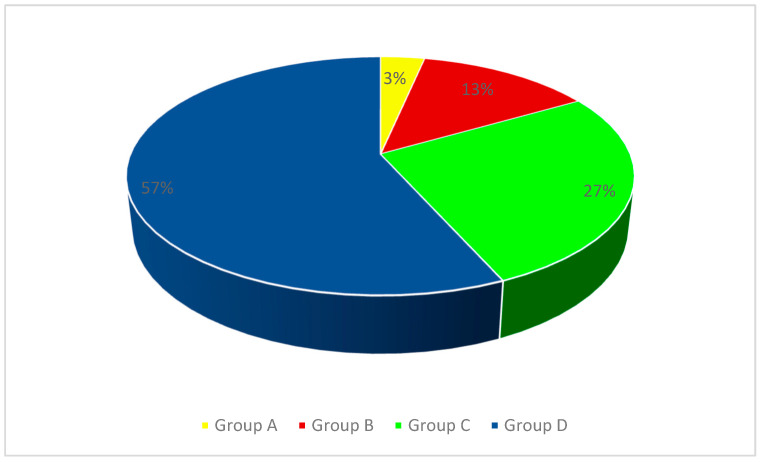
The percentage distribution of patients from individual age groups.

**Figure 2:**
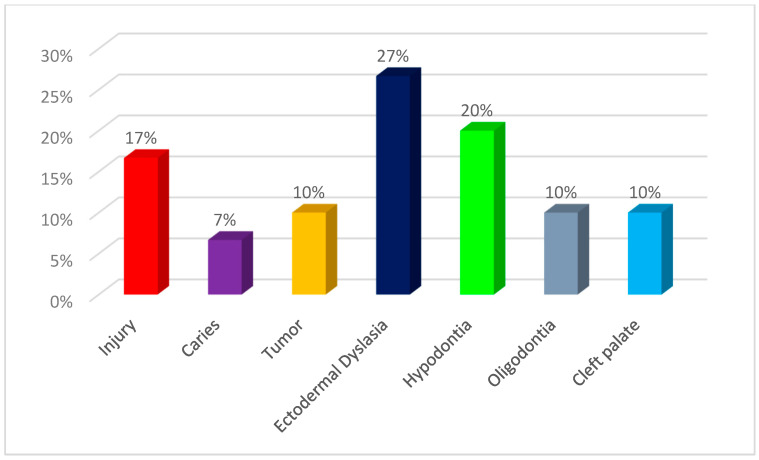
The percentage distribution of patients depending on the causes of the stomatognathic system disorders.

Due to the ages of the subjects and the degree of development of the stomatognathic system, the patients were divided into four groups. For patients of developmental age, the Carrel and Chialastri classification was used [23], which is most often used by clinicians conducting interdisciplinary treatment in children and adolescents. It divides patients with missing teeth into three age groups (A, B, C).The classification was modified by adding group D, which included patients with completed bone growth, who, due to disorders of the stomatognathic system resulting from congenital or acquired defects from an early age, were under the care of doctors of various specialties, and the completion of bone growth allowed for the use of supplements recommended in the group of adult patients. Group A included children up to 6 years of age, Group B included patients between 6 and 12 years of age, Group C included adolescents over 12 years of age, and Group D included patients with completed bone growth (young adults). The study group included patients whose abnormalities were a consequence of congenital developmental disorders, such as ectodermal dysplasia (DE), hypodontia, oligodontia, cleft lips and/or palates, as well as trauma, caries, or neoplastic processes in the facial part of the skull. The percentage distribution of patients from individual age groups is presented in Figure 1 (group A—1 patient; group B—4 patients; group C—8 patients; group D—17 patients), while the distribution of patients in the study group depending on the cause of the stomatognathic system disorder is shown in Figure 2 (group A—ectodermal dysplasia (1 patient); group B—caries (1 patient), tumor (2 patients), oligodontia (1 patient); group C—injury (2 patients), caries (1 patient), tumor (1 patient), ectodermal dysplasia (2 patients), hypodontia (1 patient), cleft palate (1 patient); group D—injury at an early age (3 patients), ED (5 patients), hypodontia (5 patients), oligodontia (2 patients), cleft palate (2 patients)).

### 2.2. Questionnaires

In the conducted retrospective studies, the quality of life of the patients was assessed with the use of the CPQ 11-14 (Child Perception Questionnaire 11-14), OQLQ (Orthognathic Quality of Life Questionnaire), and PIDAQ (Psychosocial Impact of Dental Aesthetics Questionnaire). The patients made a subjective assessment of the impact of the deformation of the stomatognathic tissues on selected psychological and social aspects, daily activity, self-esteem, and quality of life. In accordance with the recommendations for the assessment of the quality of life, a test battery was used, which is a set of several questionnaires. This procedure allowed for the assessment of a wide range of determinants influencing the quality of life in relation to the health or disease of the oral cavity. All questionnaires used contained questions classified as nominal scales. The answers were ranked in ascending order, using a five-point Likert scale, which allowed us to obtain numerical data on the frequency of occurrence or the degree of acceptance of the examined phenomenon.

The Child Perception Questionnaire 11-14 (CPQ 11-14) is intended mainly for children between 11 and 14 years of age; it contains 37 questions on the basis of which the respondents assessed the frequency of oral abnormalities (6 questions), limitations in functioning (9 questions), the impact of oral cavity disorders on their well-being (9 questions), and interpersonal contacts and behavior at school (13 questions). The respondents defined the frequency of symptoms, problems, and limitations according to a five-point scale, where 0 meant never, and 4 meant every day or almost every day.

The OQLQ (Orthognathic Quality of Life Questionnaire) and PIDAQ (Psychosocial Impact of Dental Aesthetics Questionnaire) are surveys that provide additional information on facial aesthetics, self-esteem, and social relations. The Orthodontic Quality of Life Questionnaire (OQLQ) relates directly to abnormalities in the face and mouth. The subjects assessed the impact of deformation of the stomatognathic system on several aspects, such as interpersonal contacts (8 questions), facial aesthetics (5 questions), oral cavity functions (5 questions), and awareness and perception of these disorders (4 questions). The assessment was also carried out using the Likert scale; in this case, 0 meant no problem, and 4 meant a big problem.

The PIDAQ Questionnaire for Psychosocial Aesthetics of Dental Aesthetics was used to assess the impact of certain parameters of cosmetic dentistry on the psychosocial quality of life in young adults (between 18 and 30 years of age). The form contained 23 statements or phrases concerning the influence of the condition of the oral cavity and the aesthetics of teeth in relation to four categories, such as self-esteem regarding “dental” self-confidence (6 statements), social contacts (8 statements), psychological aspect (6 statements), and concerns of aesthetics (3 statements).

The questionnaire research was conducted twice—once before and once after the prosthetic rehabilitation. The aim of the first stage of the questionnaire research was to obtain information regarding to what extent the deficit in the oral cavity affects the studied aspects of life; the treatment needs in the fields of prosthetics and aesthetics and the psychological and social aspects were also analyzed. In the second stage, it was examined whether the assessment of selected quality of life parameters had an influence on the reconstruction of the dental arches with the replacement of missing tissues and the aesthetic reconstruction of the stomatognathic structures, ensuring the norms of correct occlusion, the restoration of the correct spatial position of the mandible in relation to the jaw, and the functional rehabilitation of the masticatory organ.

### 2.3. Statistical Analysis

The values of the sums of individual questionnaires before and after prosthetic rehabilitation were subjected to statistical analysis. The data were evaluated for normal distribution using the Shapiro–Wilk test. This test confirmed the normal distribution in all individual study groups; then, Student’s *t*-test was used for dependent samples for the significance level of *p* ≤ 0.05. All data were computed using the Statistica 12.0 program (StatSoft, Inc., Tulsa, OK, USA). On the basis of the nature of the results achieved, anormal distribution of the samples was confirmed by a strong Shapiro–Wilk test, and due to the possibility of using the parametric *t*-test, the authors decided not to provide a sample size calculation and to perform a statistical analyses with 30 fully filled questionnaires.

## 3. Results

When assessing the quality of life on the basis of individual questionnaires, the patients could obtain, respectively, 0 to 148 points for the CPQ 11-14 questionnaire, 0 to 88 points in the OQLQ form, and 0 to 92 points in the PIDAQ. The assessments were made according to the Likert’s scale, where a high numerical value confirmed poor oral health and low quality of life. Only in the PIDAQ form in the first part of the questionnaire (questions 1–6) were the patients assessed positively, i.e., the higher the numerical value, the better the self-assessment of the dental appearance.

**Figure 3:**
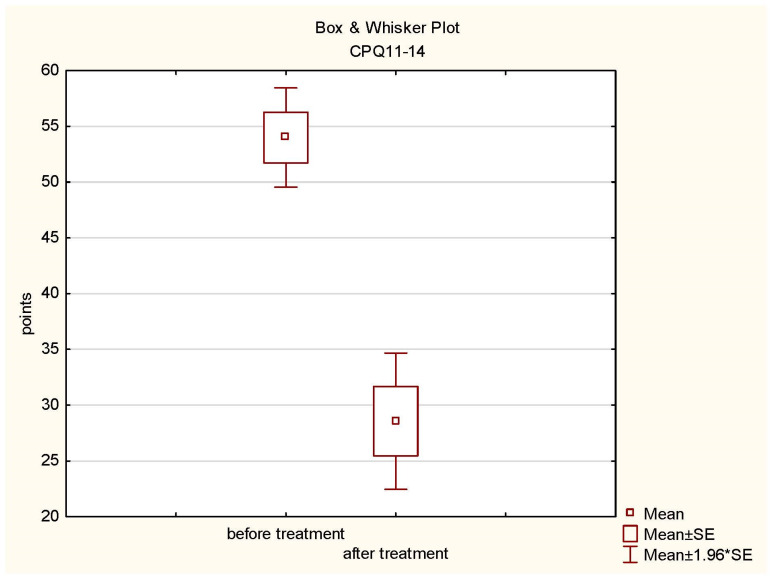
Box plot of CPQ 11-14 before and after treatment.

Before the prosthetic treatments, the highest values in the individual questionnaires were, respectively, CPQ 11-14—94 points; OQLQ—64 points; and PIDAQ—68 points. The following values were obtained after the treatments: CPQ 11-14—60 points; OQLQ—48 points; and PIDAQ—44 points. A statistical analysis was performed on the data obtained before and after prosthetic rehabilitation in the CPQ 11-14 Child Perception Questionnaire. Student’s *t*-test was used for the dependent samples for the significance level of *p* ≤ 0.05. The SUMA CPQ11-14 study showed a statistically significant difference in the assessment of patients before (mean = 54; standard deviation = 12.43) and after the treatments (mean = 28.57; standard deviation = 17.04) (*t*-test values = 10.2895479, *p* = 0.00). The results are depicted in the diagram below (Figure 3).

**Figure 4:**
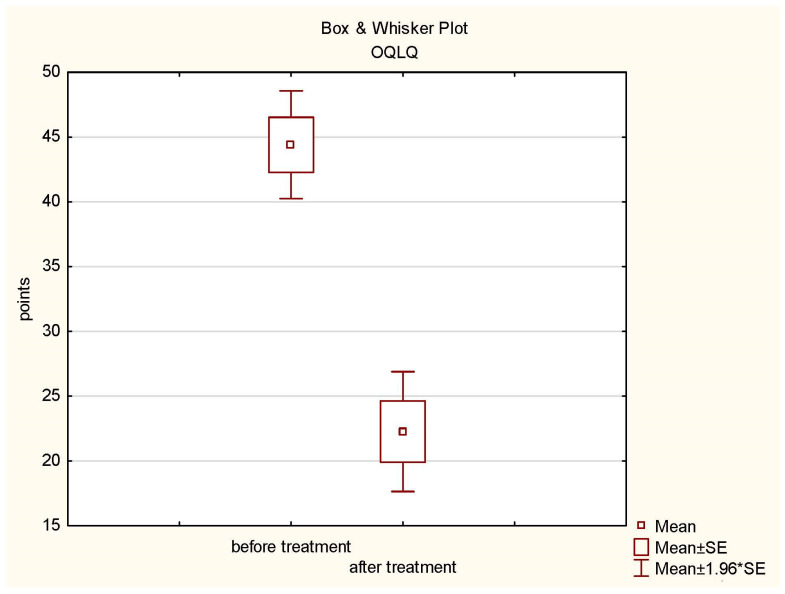
Box plot of OQLQ before and after treatment.

A statistical analysis of the results of the Orthognathic Quality of Life Questionnaire (OQLQ) was performed. Student’s *t*-test was used for the dependent samples for the significance level of *p* ≤ 0.05. The OQLQ study showed a statistically significant difference in the assessment of patients before (mean = 44.4; standard deviation = 11.63) and after treatment (mean = 22.27; standard deviation = 12.91) (*t*-test values = 9.229346, *p* = 0.00). The results are presented in the diagram below (Figure 4).

**Figure 5:**
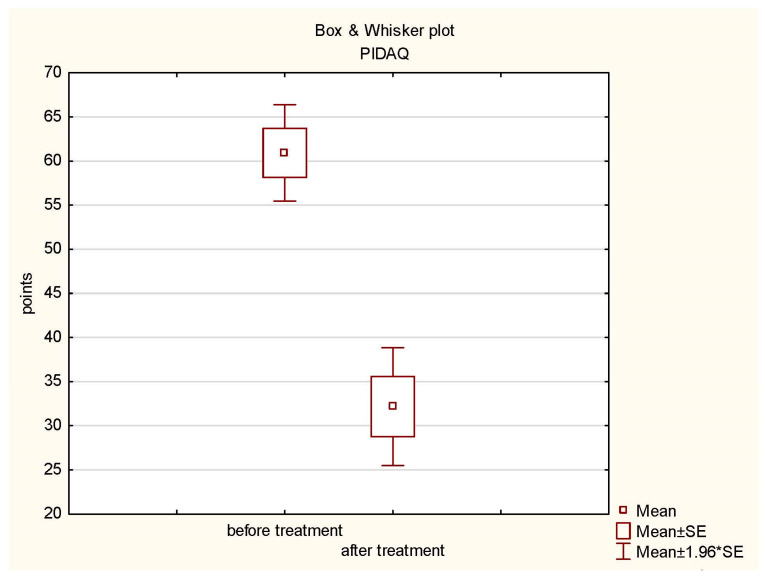
Box plot of PIDAQ before and after treatment.

In the statistical analysis of the results of the PIDAQ (Questionnaire of the Psychosocial Impact of Dental Aesthetics), Student’s *t*-test was applied for the dependent samples with the significance level of *p* ≤ 0.05. The SUMA PIDAQ study showed a statistically significant difference in the assessment of patients before (mean = 47.6; standard deviation = 8.67) and after the completion of interdisciplinary rehabilitation (mean = 34.03; standard deviation = 6.71) (*t*-test = 6.79960281, *p* = 0.00). The results are presented in the diagram below (Figure 5).

**Figure 6:**
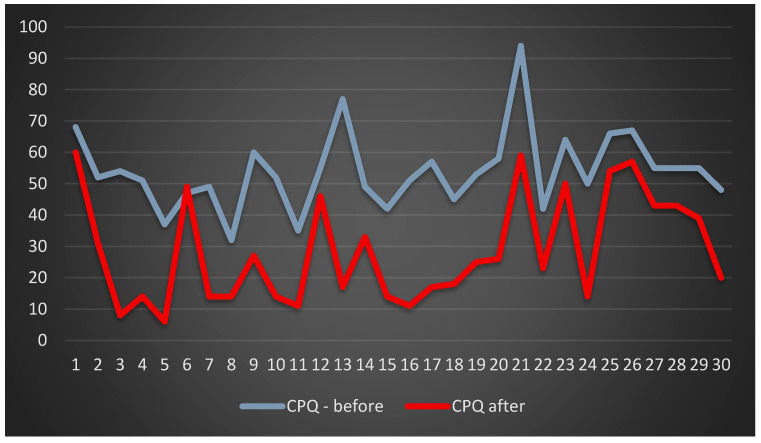
Patients’ quality of life assessment on the basis of the CPQ 11-14.

The total numerical results obtained in the CPQ 11-14, except for one case, decreased, which means that prosthetic rehabilitation had a positive impact on the assessment of the quality of life regarding individual categories (Figure 6).

**Figure 7:**
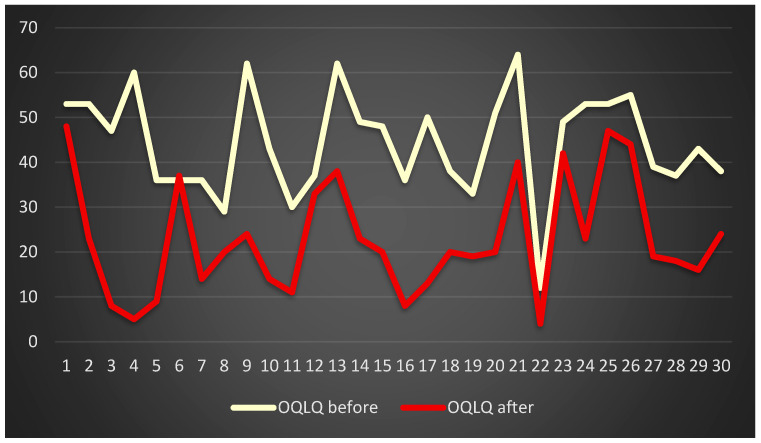
Patients’ quality of life assessment on the basis of OQLQ.

**Figure 8:**
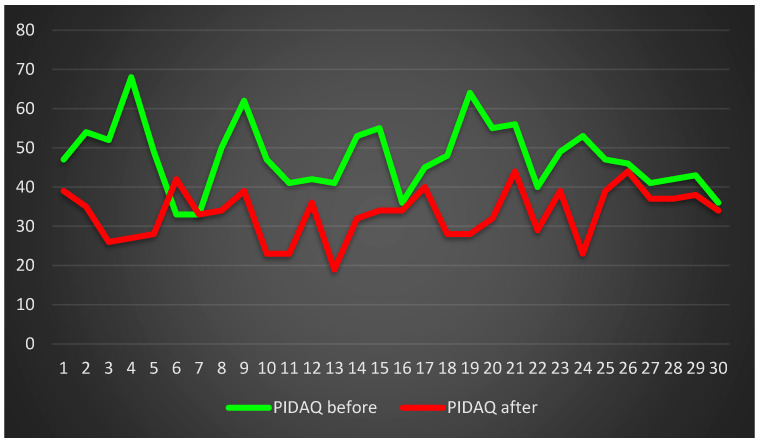
Patients’ quality of life assessment on the basis of PIDAQ.

Similar results were obtained in the OQLQ (Figure 7) and the PIDAQ (Figure 8).

## 4. Discussion

Prosthetic rehabilitation for children and juvenile patients with craniofacial disorders is a multi-specialist treatment, and due to the different ages of patients and the etiology and specificity of the disorders, it is carried out in many stages. Extremely important issues are the multifaceted diagnosis of the patient in terms of morphological and functional disorders and the impact of malformation on the development of the stomatognathic system and the well-being of an adolescent patient.

The impact of these disorders on a patient’s daily functioning, self-esteem, and psyche is also of great importance. This is confirmed by the research conducted by McGrath et al. and Warschausky et al. [24,25]. For this reason, it is necessary to diagnose a patient in order to determine the optimal method of treatment, the sequence of applied procedures, and the verification of the impact of therapy on the assessment of the patient’s quality of life.

The treatment plan should include an interdisciplinary assessment of the stomatognathic system supplemented with data from patients, focusing on their interpersonal problems, oral function disorders, and psycho-social limitations. Three types of questionnaires (CPQ11-14, OQLQ, and PIDAQ) were used to identify the factors affecting an individual’s health, to plan and monitor the treatment, assess the impacts of different treatment regimens, analyze the changes in individual patients over time, and improve patient–physician communication, according to applications proposed by Robinson et al. [26]. In the case of the treatment of patients in different age groups with various disorders and different therapeutic solutions, it is difficult to obtain consistency in the OHRQol measurements to compare the studied groups or population. In the study, after multi-stage interdisciplinary rehabilitation, including prosthetic rehabilitation, an attempt was also made to assess the level of quality of life in children and adolescents with developmental disorders in the facial part of the skull before the planned treatment and after the completion of therapy. This study analyzed the influence of disorders on parameters of the quality of life such as widely understood disorders of the oral cavity function, the perception of one’s appearance, self-esteem, and interpersonal contacts.

The patients completed the forms in the correct order, depending on the subject of the surveys. They started with the CPQ 11-14, in which they assessed the incidence of irregularities within the mouth, restrictions in functioning, the impact of oral disorders on their well-being, interpersonal contacts, and their behavior at school. The numeric values for the individual questions were summed to evaluate both the individual categories and the total value of the CPQ 11-14 form. Overall, the higher the number, the worse the oral health assessment was. Another questionnaire was the OQLQ. It was a shorter survey in a form that provided additional information on the facial aesthetics, self-esteem, and oral disorders. The questions concerned similar topics, but their shorter form and differently formulated questions could change the patients’ views on a given problem. The third PIDAQ survey was mainly about the assessment of the impact of specific parameters of aesthetic dentistry and their disorders on the quality of life in the psychosocial aspect. The researchers also sensitized patients for the first part of this survey, where the assessment, unlike the other domains of all surveys, concerned the positive feelings of the patient. The order of surveys was gradually transferred to the accent in the assessment of disorders in the functioning of the dental system to the aesthetics and psychological and social feelings. At the same time, in the case of a dentist who often does not have sufficient psychological skills, systematized questions in the field of self-esteem or psychosocial feelings are very helpful tools in conversation with a patient. The numerical values introduced by patients with individual questions emphasize the type and importance of the problem.

Taking into consideration the ages of the surveyed patients and the percentage distribution in individual age groups, it should be emphasized that in group A (children up to 6 years of age), it is very difficult to obtain reliable results from the questionnaires, because the patients are too young to read the questions themselves. In this group, the answers given are a combination of the subjective assessment of the parent and the child. The study conducted by Wilson-Genderson et al. [27] showed a difference in the COHIP surveys between children and carers, especially in patients with disorders on the deformation of the facial part of the skull. The turnout was also a problem. Only 10% of parents in this group, mainly mothers, were so strongly involved in the process of treating their children that they expressed their willingness to cooperate and positively treated the request for help in the survey. This does not confirm the results of other authors who have obtained a share in the OHRQOL study at 74% [28]. Bearing in mind the fact that many authors emphasize the early initiation of treatment in patients with congenital defects and problems in the adaptation of young patients to the therapeutic team and treatment procedures, any information on the studied domains of quality of life is extremely important. The observations of parents, children, and doctors are very helpful in developing effective algorithms for the management of child–parent–doctor cooperation. Also, patients between the ages of 6 and 12 seem too young to read and complete quality of life questionnaires with full understanding. The solution may be to introduce a short, simple questionnaire with clear pictorial elements for the youngest age group or a shortened version of the OHIP-49 questionnaire, i.e., the OHIP-14 questionnaire, for patients in group B. The studies by Jokovic et al. and Allen et al. [29,30] on the validity and reliability of the shorter forms of questionnaires show that they can be used as simpler tools in clinical conditions to analyze the results on a general scale. On the other hand, if the sensitivity of measurement and discriminant properties with regard to individual domains are considered, then the use of the original version of the questionnaires is more justified [31,32]. A subjective assessment and the “discretion” of some responses turned out to be problems in the survey-based research. The introduction of several questionnaires and a careful analysis of the results concerning the homogeneous subject matter of the questions in different questionnaires showed differences in the answers to very similarly formulated questions, although the questionnaires were completed at the same time. This problem was raised by other authors, who noticed that respondents became distracted with questionnaires that were too long and extensive. [33,34,35]. The patients filled the OQLQ questionnaire last, and it was seen in the results that 9 out of 30 patients marked different answers in the point of the smile in the OQLQ and PIDAQ. This is confirmed by the considerations in the studies by Jokovic et al. or Locker and Allen [29,30] regarding the methods of creating quality of life questionnaires in relation to their statistical analysis and clinical aspects.

A statistical analysis of the results obtained in individual questionnaires before and after the prosthetic treatment (or after the completed stage of prosthetic treatment adequate to the patient’s age) confirms the positive impact of prosthetic rehabilitation among patients in all study groups. The obtained results confirm the improvement of the quality of life of the treated patients in relation to all of the tested quality of life parameters, such as functional limitations, pain, psychological problems, limitations, or physiological, mental, and social disorders. In the case of multi-stage treatment, standardized quality of life questionnaires also make it possible to compare patients’ assessments at individual stages of treatment with regard to the type of supplements used and the dynamics of changes in relation to a patient’s age and psychosocial development (change of school, environment, maturation, and first relationships). It is equally important to be able to measure the impact of treatment on a patient’s psyche and improve their quality of life, especially in the group of patients with congenital anomalies such as ectodermal dysplasia or cleft lip and palate, manifesting disorders in the appearance of juvenile patients. The studies by Anweig et al. and Hashem et al. [8,36] carried out among patients of different ages in the group of patients with hypodontia confirm low self-esteem, poor assessment of external appearance, and problems in interpersonal contacts. Moreover, about 35% of children with craniofacial malformations are shy, have many complexes, and suffer from low self-esteem. These patients, not accepting the disease, display aggressive behavior, and they are characterized by disobedience and impulsive behaviors. Considering the psychological aspect of treatment, it should be emphasized that, often, the therapy in group C falls during the period of puberty and “teenage rebellion”, which may interfere with cooperation. An analysis of the psychological well-being or self-esteem of these patients provides information that is helpful in diagnosing emotional problems or motivation to provide treatment. The authors used OHRQol as an additional tool in the holistic diagnosis and treatment of the patients. It should be emphasized that the low indicator of patients’ participation in the study (30 out of 70 treated patients) and a large difference in the age and number of people in individual age groups are obstacles in the standardization and consistency of measurements, which are the main limitations of the study. The problem in relation to the OHRQol survey is also the long duration of the treatment, the stages associated with the development of the stomatognathic system in adolescent patients, and the complexity of interdisciplinary clinical procedures.

## 5. Conclusions

The questionnaire studies and the analysis of the obtained results showed that the assessments of pain; mental, social, and functional limitations; impairments and difficulties in everyday functioning; as well as the sense of social well-being and the impact of dental aesthetics and facial deformity on the self-esteem of patients before and after prosthetic treatment were different, which means that the treatment had a positive effect on the assessment of the above-mentioned quality of life parameters. Holistic patient rehabilitation assumes therapy that leads to the reconstruction of structures, improving the activities of the dental system, but it also provides the patient with acceptable mental and physical well-being, and helps them accept their body and appearance. The use of measurable methods to assess the impact of the disease and treatment on the quality of life of a patient is helpful in the holistic rehabilitation of patients. Survey-based research allowed us to conduct a multi-level assessment of patients’ problems, select therapy, and attempt to implement adequate prosthetic solutions.

## Data Availability

The data that support the findings of this study are available from the corresponding author upon reasonable request.
